# Correlation of Hemoglobin A1c With Wagner Classification in Patients With Diabetic Foot

**DOI:** 10.7759/cureus.9199

**Published:** 2020-07-15

**Authors:** Umar Farooque, Ashok Kumar Lohano, Sadam Hussain Rind, Muhammad Saleem Rind, Sundas Karimi, Ali Jaan, Farah Yasmin, Omer Cheema

**Affiliations:** 1 Neurology, Dow University of Health Sciences, Karachi, PAK; 2 Medicine, Peoples University of Medical and Health Sciences for Women, Nawabshah, PAK; 3 Internal Medicine, Peoples University of Medical and Health Sciences for Women, Nawabshah, PAK; 4 Medicine, College of Physicians and Surgeons Pakistan, Karachi, PAK; 5 General Surgery, Combined Military Hospital, Karachi, PAK; 6 Internal Medicine, King Edward Medical University, Mayo Hospital, Lahore, PAK; 7 Cardiology, Dow University of Health Sciences, Karachi, PAK; 8 Internal Medicine, Dow University of Health Sciences, Karachi, PAK

**Keywords:** hba1c, diabetic foot, wagner classification, positive correlation, human, diabetes mellitus, complications

## Abstract

Introduction

Diabetic foot is a common complication of diabetes mellitus (DM). The Wagner classification is mostly used to grade its severity. The correlation between the hemoglobin A1c (HbA1c) and the Wagner classification is still controversial. Therefore, the purpose of this study is to determine the correlation of HbA1c with Wagner classification in patients with diabetic foot.

Materials and methods

This cross-sectional study was conducted at a major hospital in Shaheed Benazirabad in which 88 patients aged 18-65 years, of either gender, with a known history of DM type I or type II, and diagnosed with diabetic foot were enrolled for six months. Blood samples were collected to check the HbA1c levels. Wagner classification grading was performed after the examination of diabetic foot ulcers. Demographics such as age, gender, duration of DM, and other risk factors of foot ulcers were also noted. The mean and standard deviation for continuous variables, such as age and HbA1c level, and the frequency and percentage for categorical variables, such as distribution of age, distribution of HbA1c, gender, duration of DM, grades of Wagner classification, and other risk factors of foot ulcers, were calculated. The correlation of HbA1c with Wagner classification was also calculated by applying the chi-square test and taking p ≤ 0.05 as significant.

Results

The mean age of the study population was 47.4 ± 10.6 years. Of the 88 patients, 15 (17.04%) were 25-35 years of age, 34 (38.63%) were 36-50 years of age, and 39 (44.31%) were 51-65 years of age; 45 (51.13%) patients were males and 43 (48.86%) patients were females. The mean HbA1c level of the study population was 9.07 ± 1.65%; 5 (5.68%) patients had 6.5-7.5%, 34 (38.63%) patients had 7.6-8.5%, 24 (27.27%) patients had 8.6-9.5%, and 25 (28.41%) patients had an HbA1c level of >9.5%. Twelve (13.63%) patients had ≤ 7 years, 18 (20.45%) had 8-15 years, and 58 (65.9%) had >15 years of duration of DM. Zero (0%) patients had grade 0, 1 (1.13%) patient had grade 1, 6 (6.81%) patients had grade 2, 29 (32.95%) patients had grade 3, 32 (36.36%) patients had grade 4, and 20 (22.72%) patients had grade 5 of Wagner classification. 23 (26.13%) patients had foot abnormalities, 19 (21.59%) patients had nephropathy, 13 (14.77%) patients had neuropathy, 14 (15.91%) patients had hypertension, 9 (10.22%) patients had retinopathy, 3 (3.41%) patients had foot ulcers/toe amputation, 2 (2.27%) patients had a cognitive deficit, and 5 (5.68%) patients had cardiovascular diseases. The correlation of HbA1c with Wagner classification was found statistically significant with p < 0.00001.

Conclusions

The older age, male gender, longer duration of DM, increased HbA1c, and previously existing foot abnormalities in diabetic patients are the risk factors of diabetic foot. The monitoring of HbA1c can help predict the diabetic foot in the aforesaid high-risk diabetics because the HbA1c linearly rises with the higher grades of Wagner classification of diabetic foot. Subsequently, the strict control of HbA1c as well as patient education about proper foot care can help prevent diabetic foot and its complications. However, more studies on larger scales are needed to establish the factual relationship between HbA1c and Wagner classification.

## Introduction

The diabetic foot has a prevalence of 4% to 10%, with an annual incidence of 1% to 4.1% in patients with diabetes mellitus (DM). All ethnic groups have a 25% lifetime prevalence of diabetic foot [1].

The most frequently used classification system for the diabetic foot that evaluates ulcer depth and bone involvement is the Wagner classification, which aids in the execution of a proper treatment plan and estimation of the possible outcomes. The other, not usually applied, classification systems assess ischemia, neuropathy, and the degree of infection [2,3].

The frequency of diabetic foot ulcers in different regions of the foot and the grades of Wagner classification has already been defined. Aamir et al. stated that diabetic foot ulcers were at the forefoot in 59%, midfoot in 25%, and hindfoot in 16% of patients [4]. Hasan et al. determined the frequency of grades of Wagner classification and showed that 5.5% of patients had grade 1, 30% had grade 2, 20% had grade 3, 33.3% had grade 4, and 11.1% had grade 5 ulcers [5]. Ashraf et al. stated that 74% of patients had grade 2 and grade 3 ulcers, whereas 24% of patients had grade 5 ulcers. They also found that 75% of patients were cured with limb salvage, whereas 25% could not recover without limb amputation. They also established that 22.6% to 47% of these ulcers were neuropathic, 59% were neuro-ischemic, and 18.3% were ischemic in nature [6].

The current literature is debatable about the correlation between hemoglobin A1c (HbA1c) levels and the Wagner classification. Therefore, our study is aimed at determining the correlation of HbA1c with different grades of Wagner classification in patients with diabetic foot.

## Materials and methods

Study design and sampling

This cross-sectional study was conducted at Peoples Medical College Hospital, Shaheed Benazirabad, from October 10, 2019, to April 10, 2020 (for six months). Non-probability consecutive sampling technique was used. Through Raosoft sample size calculator, the sample size came out to be 88 patients by taking a confidence interval of 95% and a prevalence of 25%, and keeping the margin of error at 9%. Inclusion criteria are age 18-65 years, either gender, a previously diagnosed DM type I or type II, and a diagnosed diabetic foot ulcers. Young patients who had insulin-dependent diabetes were classified as having type I DM, and middle- to old-aged patients who were on oral hypoglycemic drugs were classified as having type II DM. Exclusion criteria excluded patients having liver malignancy, end-stage renal disease, co-existing viral infection (such as hepatitis B), pregnancy, traumatic ulcers, vascular disorders, Buerger’s disease, and peripheral vascular disease, or using interferon therapy.

Data collection

Patients presenting to the outpatient department or admitted to the hospital and meeting the inclusion/exclusion criteria were enrolled in this study. The pros and cons of the study were explained, and informed consent was obtained. A blood sample was collected and sent to the institutional laboratory for the HbA1c level analysis. Grading of the diabetic foot was performed after examination of the wound using Wagner classification, as shown in Table 1 [7].

**Table 1:** Wagner ulcer classification system

Grade	Lesion
0	No open lesions; may have deformity or cellulitis
1	Superficial diabetic ulcer (partial or full thickness)
2	Ulcer extension to the ligament, tendon, joint capsule, or deep fascia without abscess or osteomyelitis
3	Deep ulcer with abscess, osteomyelitis, or joint sepsis
4	Gangrene localized to the portion of the forefoot or heel
5	Extensive gangrenous involvement of the entire foot

Patient demographics such as age, gender, duration of DM, and other risk factors of foot ulcers (foot abnormalities, nephropathy, neuropathy, hypertension, retinopathy, foot ulcers/toe amputation, cognitive deficit, and cardiovascular diseases) were asked by the researcher and entered in the questionnaire.

Data analysis

Data were entered and analyzed using SPSS Version 20 (IBM Corp., Armonk, NY, USA). Mean and the standard deviation were calculated for age and HbA1c level. Frequency and percentage for range of age, range of HbA1c, gender, duration of DM, grades of Wagner classification, and other risk factors of foot ulcers (foot abnormalities, nephropathy, neuropathy, hypertension, retinopathy, foot ulcers/toe amputation, cognitive deficit, and cardiovascular diseases) were also calculated. Correlation of HbA1c with Wagner classification was also calculated, the chi-square test was applied, and p ≤ 0.05 was considered as significant.

## Results

The mean age of the patients was 47.4 ± 10.6 years, as shown in Table 2.

**Table 2:** Analysis of age

	Minimum	Maximum	Mean	Standard deviation
Age (years)	18	65	47.4	10.6

**Figure 1:**
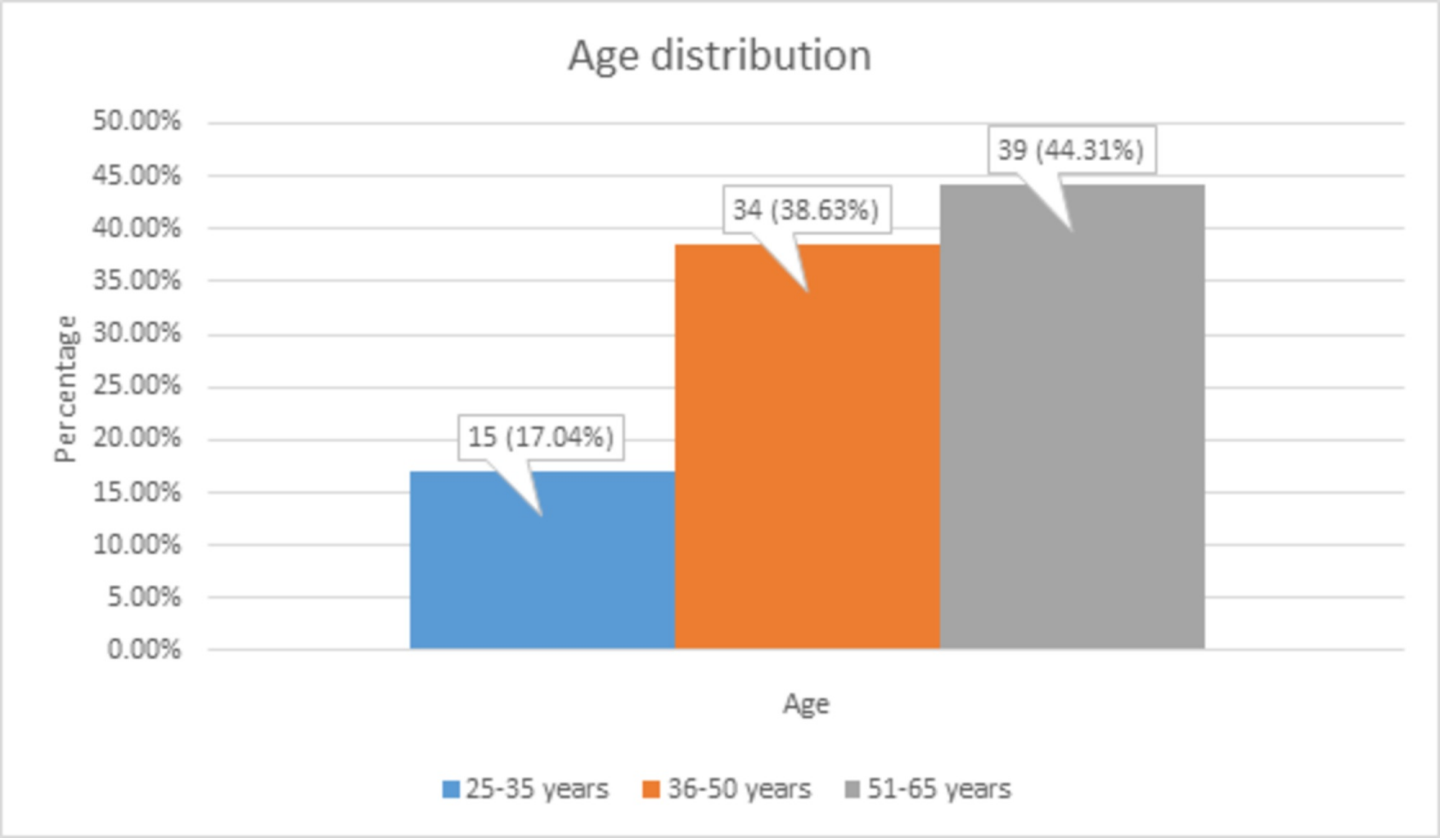
Age distribution

Of the total 88 patients, 15 (17.04%) patients aged 25-35 years, 34 (38.63%) patients aged 36-50 years, and 39 (44.31%) patients aged 51-65 years, as shown in Figure 1.

**Figure 2:**
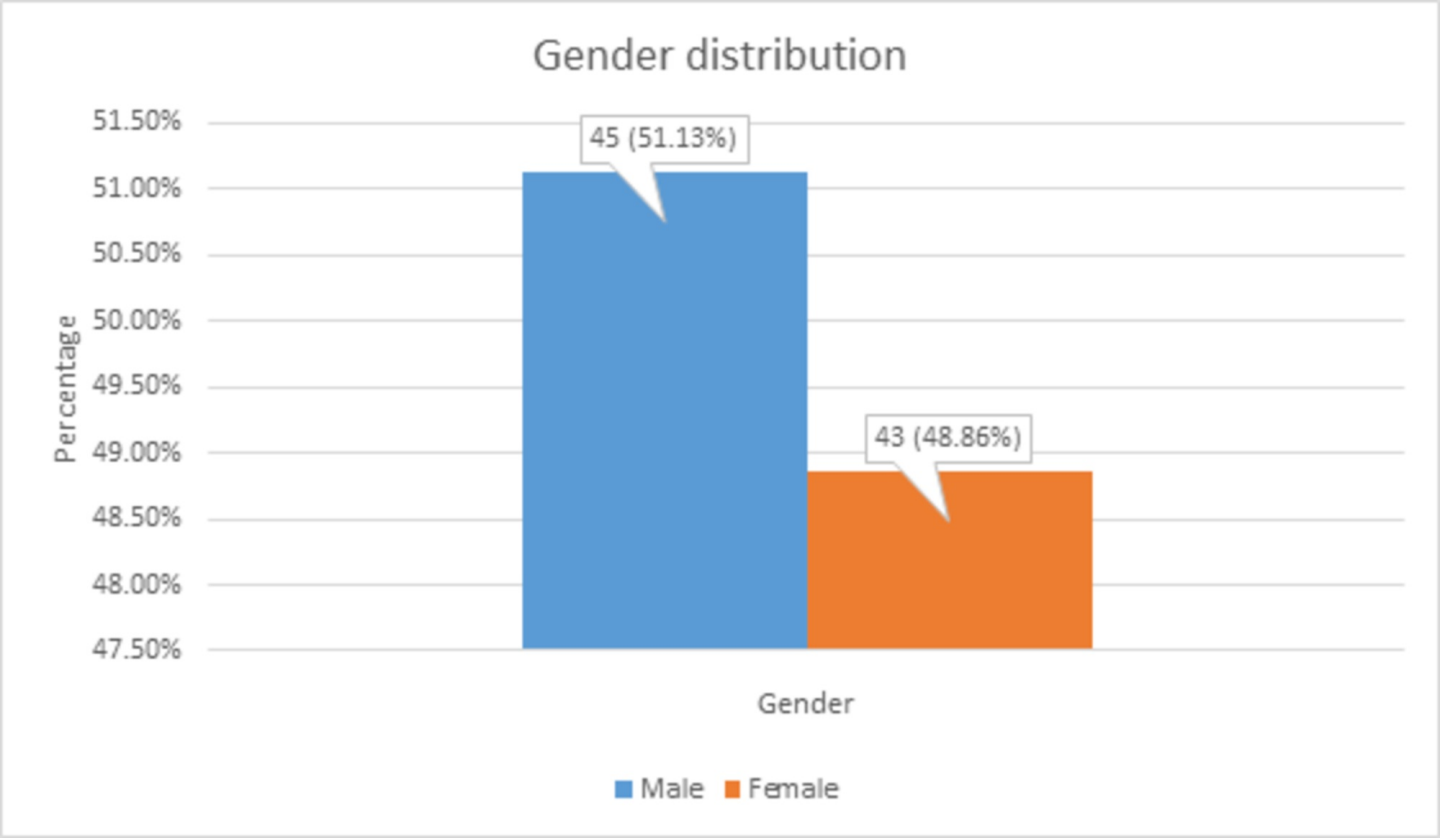
Gender distribution

There were 45 (51.13%) male and 43 (48.86%) female patients, as shown in Figure 2.

The mean HbA1c level of the patients was 9.07 ± 1.65%, as shown in Table 3.

**Table 3:** Analysis of HbA1c level HbA1c, hemoglobin A1c

	Minimum	Maximum	Mean	Standard deviation
HbA1c level (%)	6.5	11.5	9.07	1.65

**Figure 3:**
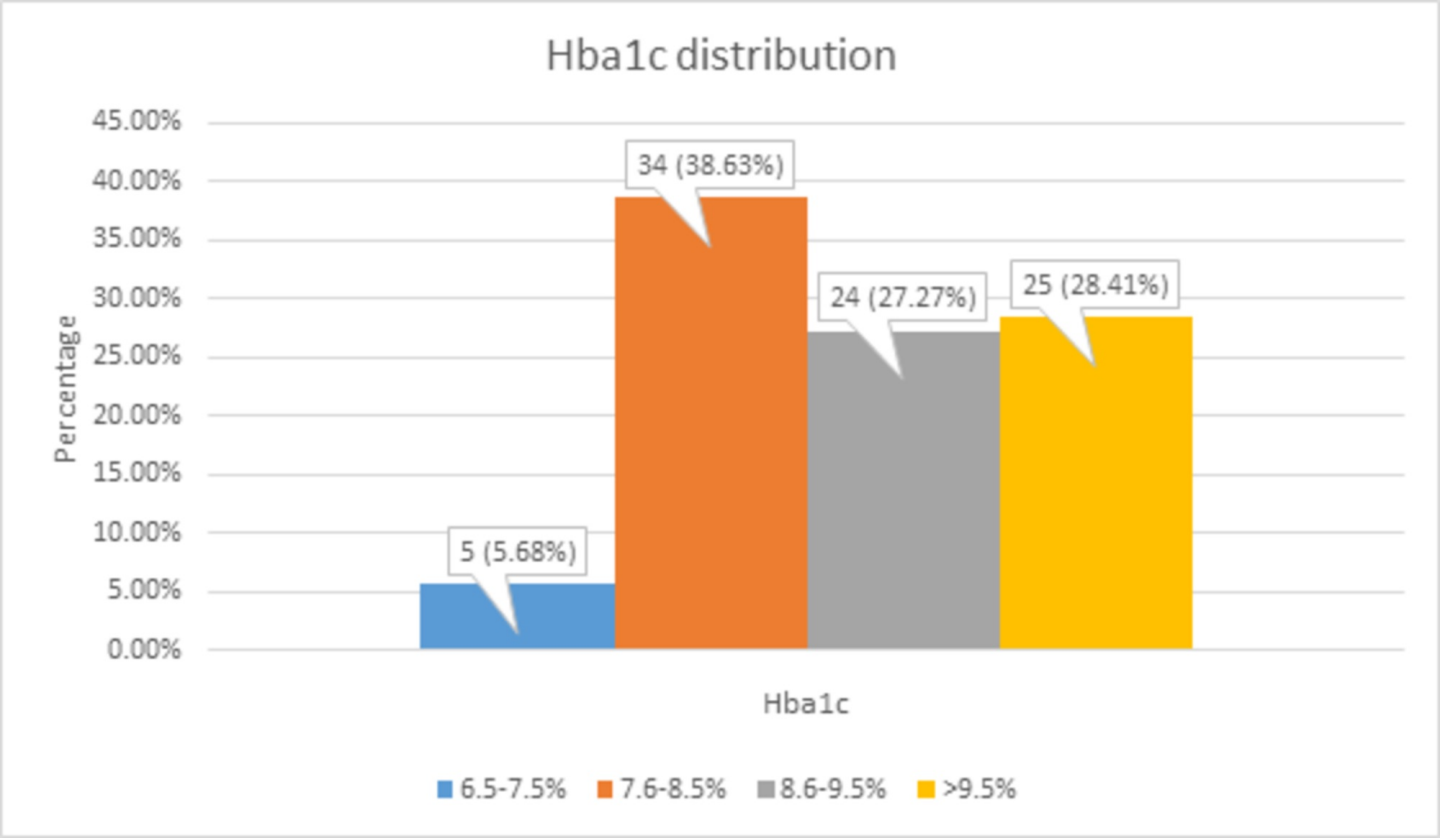
HbA1c distribution HbA1c, hemoglobin A1c

There were 5 (5.68%) patients with 6.5-7.5%, 34 (38.63%) patients with 7.6-8.5%, 24 (27.27%) patients with 8.6-9.5%, and 25 (28.41%) patients with >9.5% of HbA1c level, as shown in Figure 3.

**Figure 4:**
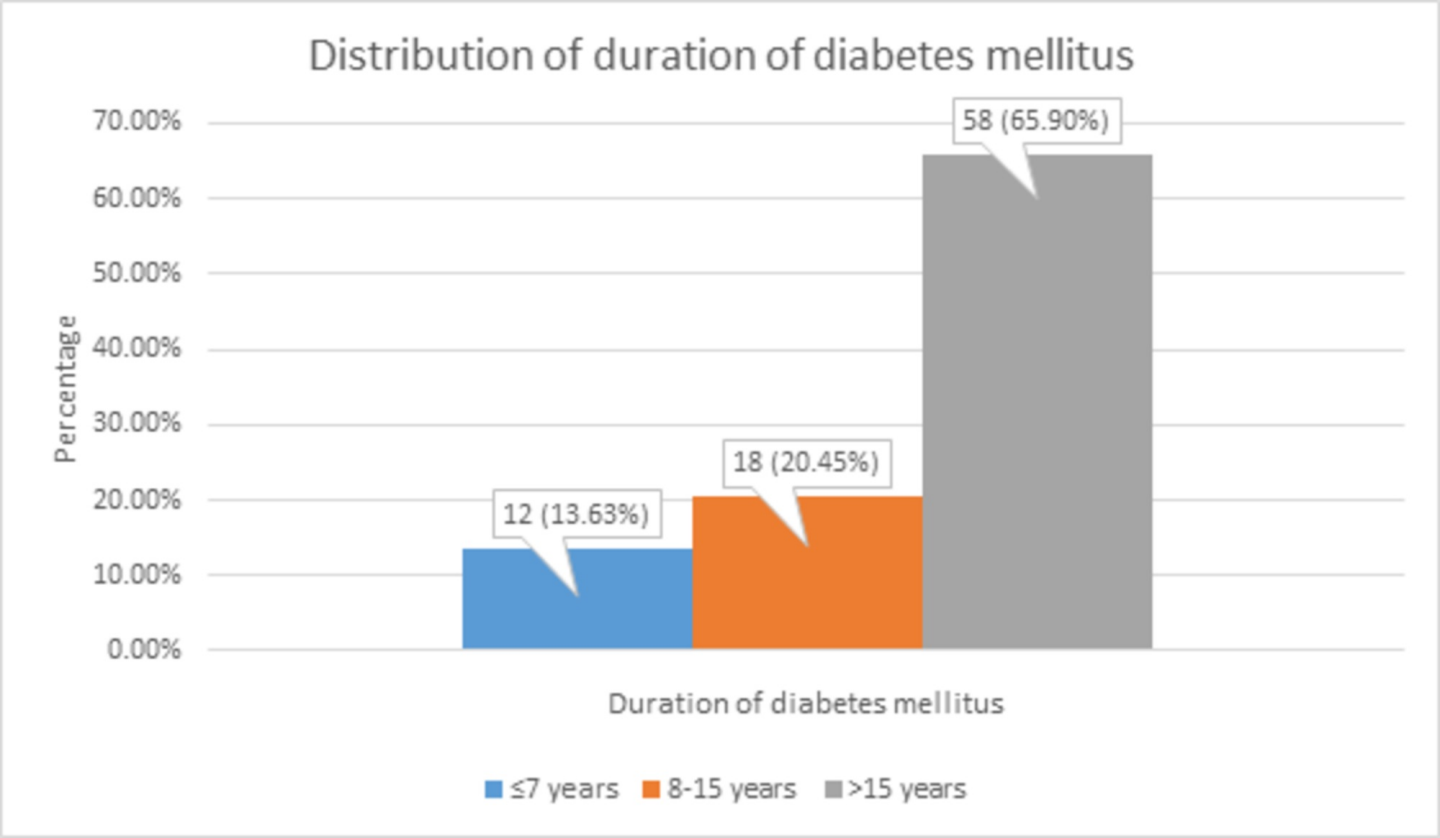
Distribution of duration of diabetes mellitus

The duration of DM was ≤7 years in 12 (13.63%) patients, 8-15 years in 18 (20.45%) patients, and >15 years in 58 (65.9%) patients, as shown in Figure 4.

**Figure 5:**
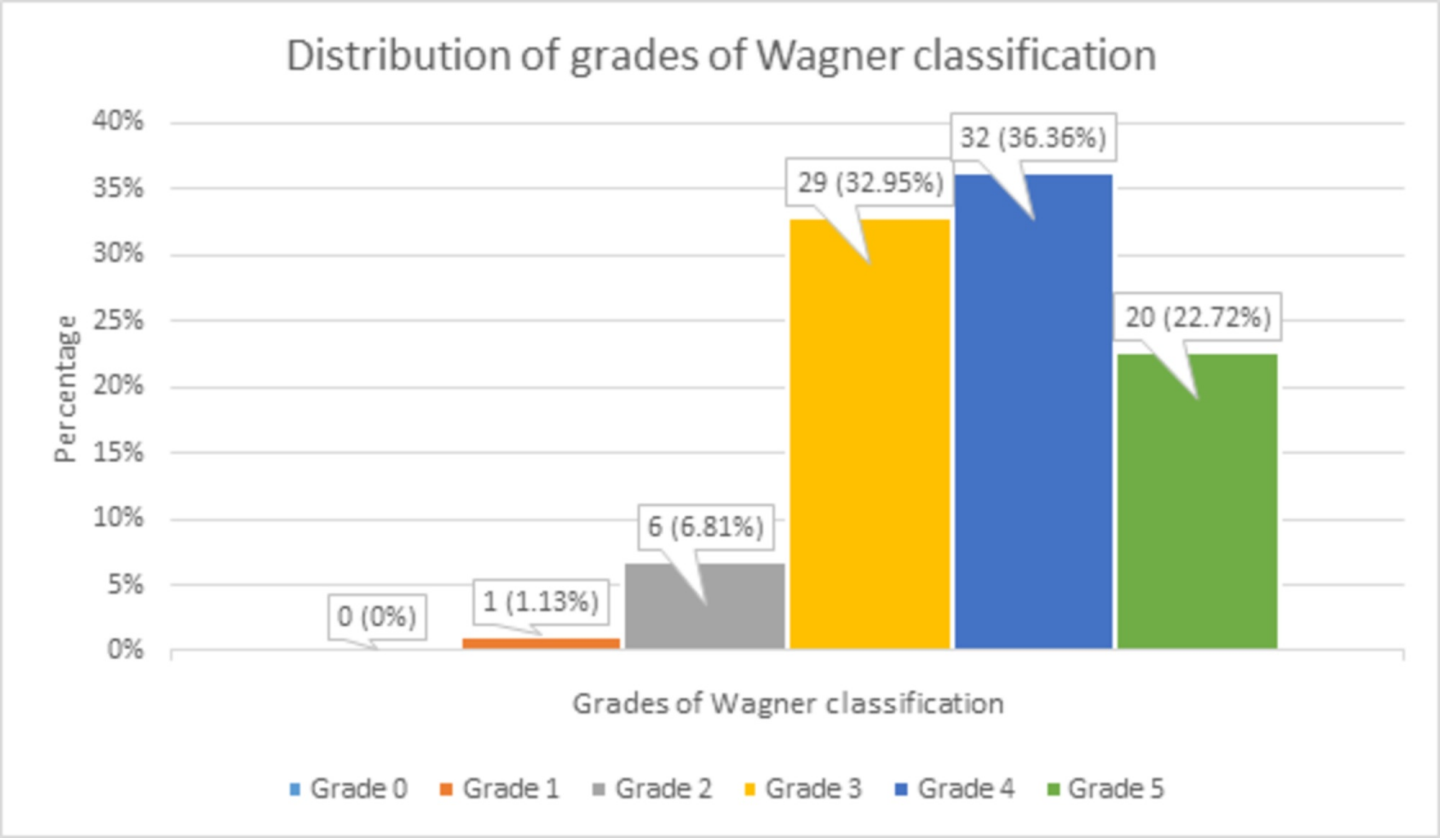
Distribution of grades of Wagner classification

There were 0 (0%) patients with grade 0, 1 (1.13%) patient with grade 1, 6 (6.81%) patients with grade 2, 29 (32.95%) patients with grade 3, 32 (36.36%) patients with grade 4, and 20 (22.72%) patients with grade 5 of Wagner classification, as shown in Figure 5.

**Figure 6:**
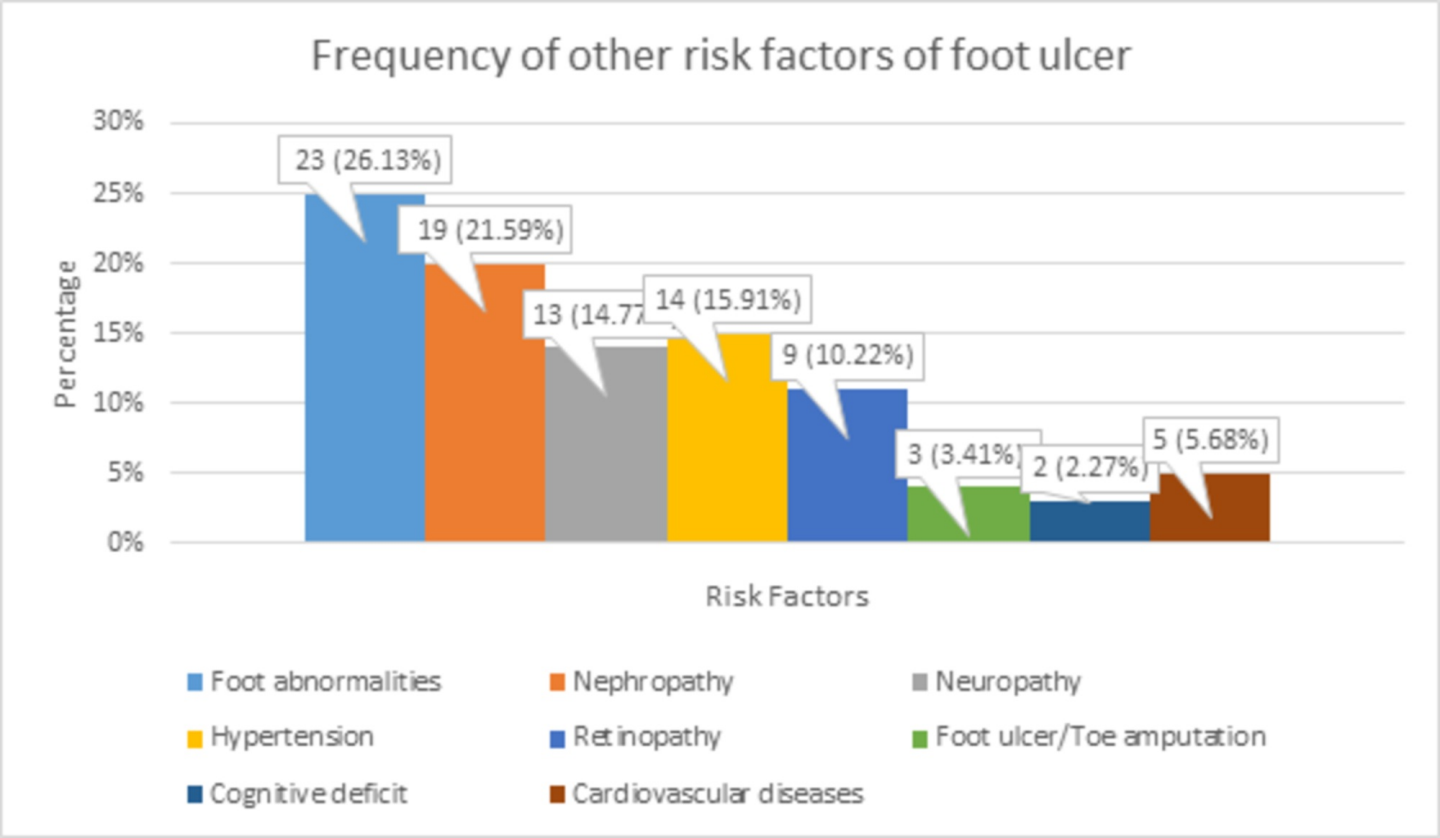
Frequency of other risk factors of foot ulcer

There were 23 (26.13%) patients with foot abnormalities, 19 (21.59%) patients with nephropathy, 13 (14.77%) patients with neuropathy, 14 (15.91%) patients with hypertension, 9 (10.22%) patients with retinopathy, 3 (3.41%) patients with foot ulcers/toe amputation, 2 (2.27%) patients with a cognitive deficit, and 5 (5.68%) patients with cardiovascular diseases. These frequencies of other risk factors of foot ulcers are shown in Figure 6.

Correlation of HbA1c with Wagner classification showed a statistically significant linear relationship with p < 0.00001. Mostly, patients with grade 4 and 5 were found to have HbA1c > 8.5%. However, patients with grades 1-3 also had HbA1c > 6.5%. These findings are shown in Table 4.

**Table 4:** Correlation of HbA1c with Wagner classification HbA1c, hemoglobin A1c

Parameter	Wagner classification					p-Value
HbA1c	Grade 1	Grade 2	Grade 3	Grade 4	Grade 5	
6.5-7.5%	1	0	4	0	0	<0.00001
7.6-8.5%	0	6	21	7	0	
8.6-9.5%	0	0	4	18	2	
>9.5%	0	0	0	7	18	

## Discussion

This study indicates that there is a linear relationship between the HbA1c level and the grades of Wagner classification. Patients classified in grades 0-2 of Wagner classification had slightly elevated HbA1c levels, whereas patients with grades 3-5 had the highest HbA1c levels mainly due to non-compliance of the patients.

Diabetic foot syndrome includes several diabetic foot pathologies such as infection, neuropathic osteoarthropathy, and diabetic foot ulcers. Diabetic foot, which is about 15% of these and is projected to grow up to 25%, is the most hazardous condition, which may lead to limb amputation [8].

Diabetic foot is caused by minor injuries that are not perceived for a long time by diabetic patients due to peripheral nerve dysfunction. Furthermore, peripheral nerve dysfunction often gets associated with peripheral arterial disease leading to the deficient blood supply to the limbs, a condition known as diabetic angiopathy, which may also cause diabetic foot. Therefore, the diabetic foot can be neuropathic, neuro-ischemic, or ischemic alone [6,9,10].

Zubair et al. and Ozenc et al. established a contradictory correlation between HbA1c level and Wagner classification. Zubair et al. found a statistically significant, whereas Ozenc et al. found a statistically insignificant correlation [11,12]. Sarinnapakorn et al. also showed that HbA1c level and fasting plasma glucose are not markedly related to diabetic foot, but about 50% of type II DM patients had an intermediate or high risk of diabetic foot. They categorized foot ulcer risk into low, intermediate, and high, each having a probability of 55.8%, 33.6%, and 10.6%, respectively. They found that the major risk factors of foot ulceration were age, duration of DM, estimated glomerular filtration rate (eGFR), dyslipidemia, hypertension, numbness, nephropathy, cardiovascular disease, abnormality of foot, anomalous sensation, pulse drop, ulcer, amputation, abnormal ankle-brachial index, and cerebrovascular accidents [13]. Likewise, 26.13% of our patients had foot abnormalities, 21.59% had nephropathy, 14.77% had neuropathy, 15.91% had hypertension, 10.22% had retinopathy, 3.41% had foot ulcers and toe amputation, 2.27% had a cognitive deficit, and 5.68% had cardiovascular diseases. Similarly, Wu et al. found 42% of patients suffering by foot anomalies, out of which 65% had hallux vagus [14].

The precautionary measures that patients should be educated about to decrease the incidence of the diabetic foot include strict glycemic control with lifestyle modification and compliance to medication, regular screening for foot ulcers, and self-examination for foot skin lesions, fungal infections, and deformities [1,15,16].

## Conclusions

The high-risk diabetic patients for diabetic foot are those with older age, male gender, longer duration of DM, raised HbA1c, and preexisting foot abnormalities. The HbA1c can be used as a screening tool in the aforementioned high-risk diabetic patients for the diabetic foot to predict its occurrence, as HbA1c has a linear relationship with the grades of Wagner classification of diabetic foot. This can help decrease the incidence of the diabetic foot and its related complications such as amputations, infections, disability, and death through tighter control of HbA1c and awareness about proper foot care because strict glycemic control decreases the neuropathic and vascular complications of DM. However, further large-scale studies are needed to find out the true association between HbA1c and Wagner classification.
